# Functional xanthophyll cycle and pigment content of a kleptoplastic benthic foraminifer: *Haynesina germanica*

**DOI:** 10.1371/journal.pone.0172678

**Published:** 2017-02-23

**Authors:** Thierry Jauffrais, Bruno Jesus, Vona Méléder, Emmanuelle Geslin

**Affiliations:** 1 UMR CNRS 6112 LPG-BIAF, Bio-Indicateurs Actuels et Fossiles, Université d’Angers, Angers, France; 2 EA2160, Laboratoire Mer Molécules Santé, Université de Nantes, Nantes, France; 3 BioISI–Biosystems & Integrative Sciences Institute, Campo Grande University of Lisboa, Faculty of Sciences, Lisboa, Portugal; Mount Allison University, CANADA

## Abstract

Some shallow water benthic foraminifera are able to retain functional chloroplasts (kleptoplasts) from their food source, i.e. diatoms. Here we assessed the functionality of the kleptoplast xanthophyll cycle (XC, i.e. the main diatom short-term photo-regulation mechanism) and we surveyed *Haynesina germanica* kleptoplast pigment composition over time and at different light regimes. Six common diatom lipophilic pigments were detected, two chlorophylls (Chl *a*, Chl *c*) and four carotenoids (fucoxanthin and by-products, diadinoxanthin, diatoxanthin and β-carotene), the same pigment profile as the diatom species frequently isolated at the sampling site. The xanthophyll cycle (XC) was functional with kleptoplast diatoxanthin (DT) content increase with concomitant diadinoxanthin (DD) decrease after short term light exposure. DT/(DT+DD) and DT/DD ratios increased significantly in specimens exposed to low light and high light in comparison to specimens maintained in the dark. Specimens placed in very low light after the light treatments reverted to values close to the initial ones, suggesting that *H*. *germanica* XC is functional. A functional XC is an indication of *H*. *germanica* kleptoplasts capacity for short-term photo-protection from photo-oxidative damages caused by excess of light. Furthermore, the pigment survey suggests that *H*. *germanica* preserved some chloroplasts over a longer time than others and that pigment content is influenced by previous light history. Finally, the current study highlighted seasonal differences, with higher pigment contents in winter specimens (27.35 ± 1.30 ng cell^-1^) and lower in summer specimens (6.08 ± 1.21 ng cell^-1^), a quantitative and qualitative composition suggesting light acclimation to low or high light availability, according to the season.

## Introduction

Benthic foraminiferal species have various particular physiological adaptations such as the capacity to store nitrate and to denitrify [1, 2] or to host endo- and ectosymbionts [3–6]. Among these adaptions developed by benthic foraminifera, some species have the ability to steal and sequester chloroplasts from diatoms and to keep them functional from days to many months [7–18]. This process, called kleptoplasty [19], has been observed in intertidal as well as deep-sea species (e.g. [7, 9]). However, kleptoplast biological functions have been little studied in benthic foraminifera [7, 17, 18].

In some intertidal kleptoplastic species such as *Haynesina germanica*, the photosynthetic machinery was found to be functional (net photosynthesis and carbon assimilation [7, 18, 20]), with kleptoplasts keeping their functionality for several days [18]. However, *H*. *germanica* kleptoplast functionality was significantly decreased after light exposition, which resulted in lower maximum photosystem II quantum efficiency and decreased oxygen production, even at low light (25 μmol photon m^-2^s^-1^, [18]); thus showing signs of low-light acclimation. Furthermore, *Haynesina germanica* has a diatom pigment signature; with the light harvesting pigments Chl *a*, *c* and fucoxanthin and the photoprotective pigments diadinoxanthin, diatoxanthin and β-carotene [18, 21]. Part of this photosynthetic machinery is thus susceptible to photo-damage after light exposure in the absence of active photo-protective mechanisms (reviewed in Muller et al. [22]) and also in the absence of the nuclear genes that encode most of the photosynthetic proteins. Therefore, kleptoplast functionality inside foreign cells is partially linked to the host’s capacity for photo-regulating light exposure and maintaining active photo-protection mechanisms [23, 24].

Kleptoplasts might avoid photo-damage either by using physiological photo-regulation mechanisms or by using their host behavioural response, e.g. sacoglossan sea slugs close their parapodia when exposed to excess light [23, 25, 26]. Benthic foraminifera could potentially migrate into the sediment [27, 28] or build cysts [29, 30] thus avoiding excessive ambient light, similarly to what is observed in microphytobenthic pennate diatoms that are capable of moving vertically in the sediment matrix as a photo-regulation mechanism [31–33]. *Haynesina germanica* vertical distribution is characterized by a clear maximum density at the sediment surface [34–36], where an access to light in the first millimetres is possible [37, 38]. However, *H*. *germanica* behaviour does not seem to be light driven [39] and therefore, *H*. *germanica* kleptoplast photo-regulation might be more dependent on other physiological photo-regulation mechanisms. In diatoms exposed to high light, the plastid photoprotective capacity, i.e. the xanthophyll cycle (XC), consists of a de-epoxidation reaction which convert the pigment diadinoxanthin (DD) into diatoxanthin (DT). This process is catalyzed by the diadinoxanthin de-epoxidase and regulated by the ΔpH which occurs during photosynthetic electron transport fluxes [40]. This reaction is also known to be reversible under low light intensity or in darkness [40], and is known to stay functional in other kleptoplastic organisms such as sea slugs [23, 41]. Little is known about *H*. *germanica* kleptoplast pigment content and functionality [18, 21] and therefore it is important to know if their XC is functional to understand the behaviour and distribution of this species in the sediment. The preservation of such physiological mechanisms after chloroplast assimilation is important to define the physiology and the biogeochemical capabilities (i.e. N and C assimilation, O_2_ production) of kleptoplastic benthic foraminifera. Thus, here we characterized kleptoplast pigment content in freshly sampled kleptoplastic foraminifera focussing on the dominant kleptoplastic species in mudflat systems: *H*. *germanica*. We focused on kleptoplasts xanthophyll cycle pigments during a short term exposition to different light regimes; and we followed kleptoplast pigment composition over time and different light intensity, during a longer time scale experiment.

## Materials and methods

### Sampling

**Fig 1:**
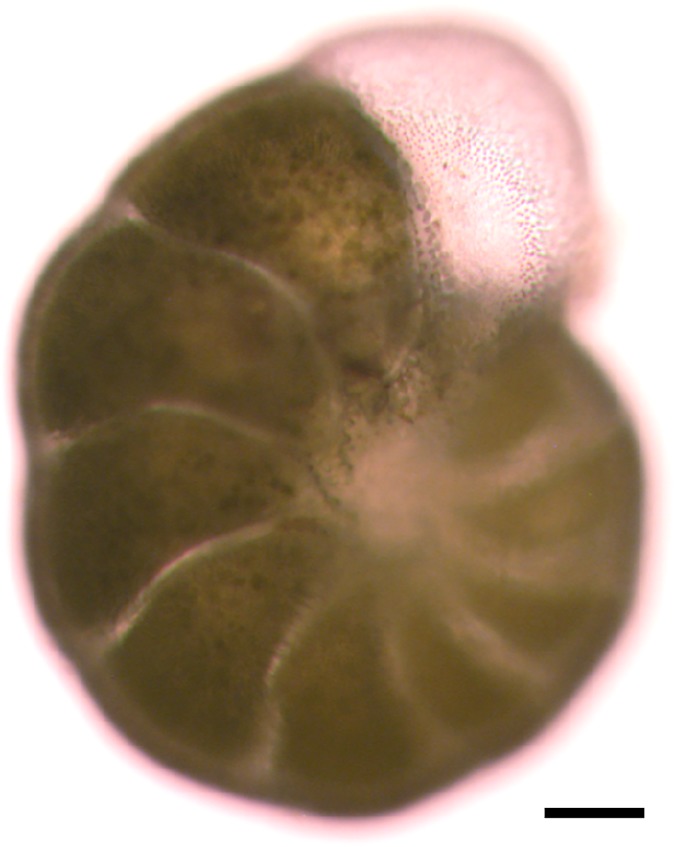
Specimen of the studied species, *Haynesina germanica*, view in light microscopy. Cell was clean with a brush, test and cytoplasm are observable, scale bar equal to 50 μm.

*Haynesina germanica* (Fig 1) specimens were sampled in July (experiment 1) and December (experiment 2) 2015 at Bourgneuf Bay (47.013°N, 2.019°W), a large mudflat on the French Atlantic coast. The upper sediment layer (first 5 mm) was sampled and sieved over 300 and 150 μm meshes using *in situ* sea-water. The 150 μm fraction was collected in dark flasks and maintained overnight in darkness at 16°C. The following day, using a stereomicroscope (Leica MZ 12.5), *H*. *germanica* in healthy conditions (i.e. with cytoplasm inside the test, e.g. Fig 1) were collected from the sediment one per one with a brush. The selected specimens were carefully cleaned with a brush and rinsed in artificial seawater (ASW) several times to minimize bacterial and microalgal contamination.

Access to the sampling site did not required any specific permissions, and the work did not involve endangered or protected species.

### Size and biovolume determination

*Haynesina germanica* shell mean maximal elongation (μm, n = 108 experiment 1, n = 60 experiment 2) was measured using ImageJ software [42] after image acquisition with a light microscope (Zeiss) coupled to a digital camera (Nikon D7000). Mean foraminiferal volume was approximated with a half sphere equation, considered the best form for *H*. *germanica* (Geslin et al., 2011). The cytoplasmic volume (or biovolume) was then estimated by assuming that internal test volume corresponds to 75% of total foraminiferal test volume (Hannah et al., 1994).

### Pigments

*Haynesina germanica* pigments were extracted in 300 μL of methanol at 95% (MeOH:H_2_O v/v) with 2% of ammonium acetate and internal standard (trans-β-Apo-8’-carotenal from Sigma Aldrich at 1 mg.L^-1^). Foraminifera were crushed and stirred with repeated strokes of a metallic row, sonicated for one minute, subsequently maintained at -20°C for 15 min, and finally filtered (0.2 μm, Whatman). The filtrate was analyzed with an Ultimate 3000 RS (Dionex) high-performance liquid chromatography (HPLC) as described in Meleder et al. [43]. Pigments were identified using their absorption spectra between 400 and 800 nm measured with the photodiode-array detector. Quantification, in ng per foraminifera cell (ng cell^-1^), was carried out at 440 nm by comparison with pigments standards (DHI, Denmark). Results are shown for major diatom pigments, chlorophylls: chlorophyll *a* (Chl *a*) and chlorophyll *c*1 + *c*2 (Chl *c*); and carotenoids: β-carotene (β-caro), fucoxanthin (Fuco) and its by-products (Fuco-like 1 and 2, 19'Butanoyloxyfucoxanthin (But-Fuco)); and xanthophyll cycle pigments (XCP = DD+DT): diadinoxanthin (DD) and diatoxanthin (DT). All pigment ratios (DT/DD, DT/ (DD+DT), (DD+DT) /Chl *a*, Chl *c*/Chl *a*, Fuco/ Chl *a*, β-caro/ Chl *a*, degraded pigments /Chl *a* and Fuco-like 1+2 / Fuco) are expressed in g g^-1^. The degradation pigments are the sum of carotenoids by-products, mainly composed of Fuco-like 1 and -2.

### Experimental set up

In the two experiments, *H*. *germanica* specimens were removed from the sediment to prevent cells from hypothetically photo-regulating by moving in the sediment matrix. In this way we avoided possible bias driven by behaviour.

#### Experiment 1

This experiment was carried out to assess xanthophyll cycle (XC) short term functionality. All experiments were carried out in a Fytoscope (FS130, Photosystem Instrument) maintained at 16°C but using different light regimes. All foraminifera specimens (n = 1500) were distributed in 15 polystyrene Petri dishes (100 per Petri dish) filled with 5 mL of ASW at 35 salinity and were “dark adapted” during one hour at a very low light (VLL) intensity (5 μmol photon m^-2^ s^-1^). VLL was chosen as a “dark adaption” methodology instead of full darkness because this treatment has been shown to work better in reversing diatom non photo-chemical quenching (NPQ) that often accumulates in darkness [44]. Initial pigment composition (T0 at VLL) was assessed after 1 h of VLL by quickly freezing with liquid nitrogen three replicates of 100 specimens in 2 mL Eppendorfs. All samples were stored at -80°C until analysis. Following the VLL adaptation period, 900 specimens were placed under three different light conditions during 30 minutes: dark (D, 3 replicates of 100 specimens), low light (LL, 25 μmol photons m^-2^ s^-1^, 3×100 specimens) and high light (HL, 300 μmol photons m^-2^ s^-1^, 3×100 specimens). A fourth treatment of 30 min HL:30 min VLL (300:5 μmol photons m^-2^ s^-1^, 3×100 specimens) was added to investigate XC reversal. All specimens were sampled and frozen as described above after their respective treatments. A HL:VLL cycle was preferred to a HL:D cycle, as with diatoms de-epoxidation driven by the chlororespiratory proton gradient might occur under dark conditions [45].

#### Experiment 2

This experiment was carried out to assess kleptoplast long-term pigment degradation over time. All measurements were made in a 16°C temperature controlled room and using different light regimes. All foraminifera specimens (n = 2100) were spread in 21 Petri dishes (100 specimens per Petri dish) filled with 5 mL of artificial sea water at 35 salinity and placed at 16°C for a “dark” adaption of one hour at VLL. Initial pigment composition (T0, 3 replicates of 100 specimens) was estimated as described above. Following dark-adaptation, 1800 specimens were placed under two different light conditions: dark (D, 9 Petri dishes of 100 specimens) and light (L, 50 μmol photons m^-2^ s^-1^, 9 Petri dishes of 100 specimens) with Light:Dark cycle of 6:18h. Foraminifera were sampled after 5, 10 and 15 days as described before, three replicates of 100 specimens in each treatment.

For both experiments (1 and 2), the light intensities applied were chosen based on experimental results obtained in our previous work (19), where *H*. *germanica* was found to produce oxygen, quickly increasing with irradiance, showing no evidence of photoinhibition within the light range used (0–300 μmol photons m^-2^ s^-1^). However, the compensation irradiance was reached very quickly, at light level as low as 24 μmol photons m^-2^ s^-1^ [18]. Furthermore, the photoperiod chosen (Light:Dark cycle of 6:18h) is based on the hypothesis that within the sediment and in turbid environments such as mudflat systems, *H*. *germanica* can have an access to light only during diurnal low tide.

### Statistical analysis

Data in tables and text are expressed as mean ± standard deviation (SD). Data in figures are expressed using box plots; the box plots shows the median, the tops and bottoms of each box mark the 75^th^ and 25^th^ percentiles and the whisker length is equal to 1.0 times the interquartile range. After testing for homogeneity of variance and normality (test Kolmogorov-Smirnov), statistical analyses consisted of analysis of variance (ANOVA) for experiment 1 and a multifactor ANOVA for experiment 2 followed by a Fisher's LSD test to compare pigment composition and ratios. Differences were considered significant at p<0.05. All statistical analyses were carried out with Statgraphics Centurion XV.I (StatPoint Technologies, Inc.) software.

## Results

### Experiment 1

*Haynesina germanica* had a mean maximal test elongation of 255 ± 32 μm (SD, n = 108). This resulted in cytoplasmic biovolumes equal to 3.26 × 10^6^ ± 6,38 × 10^3^ μm^3^ (SD).

**Fig 2:**
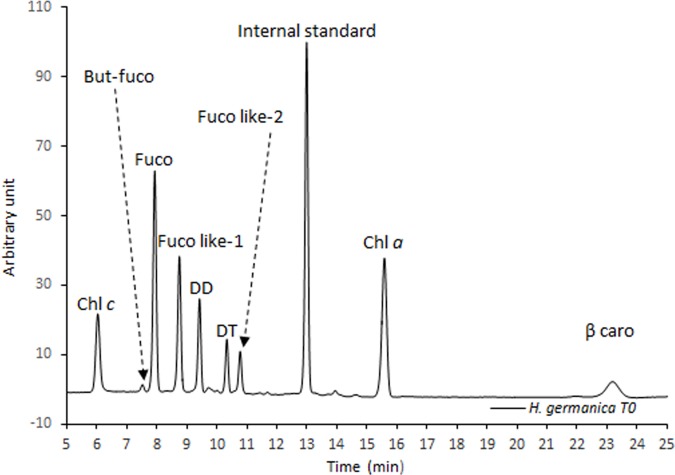
Initial (T0, experiment 1) high performance liquid chromatograms at 440 nm of pigments extracted from *Haynesina germanica*.

**Fig 3:**
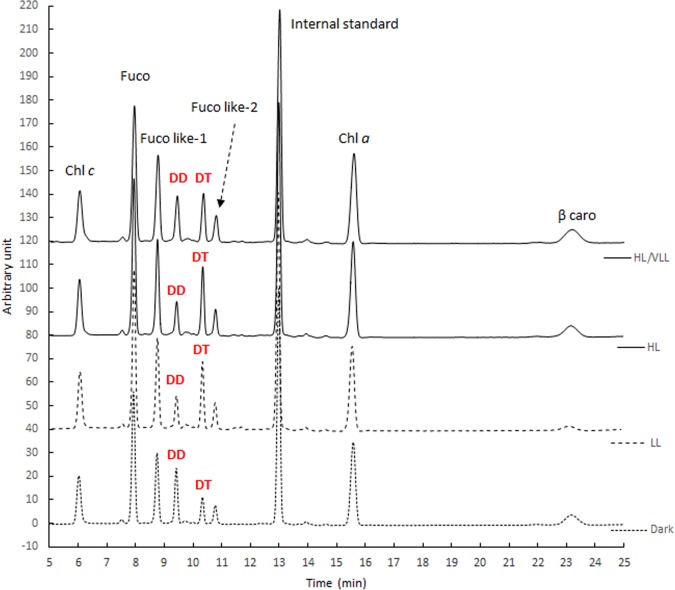
High performance liquid chromatograms at 440 nm of pigments extracted from *Haynesina germanica* after exposure to different light levels (D, LL, HL, HL/VLL, experiment 1).

**Fig 4:**
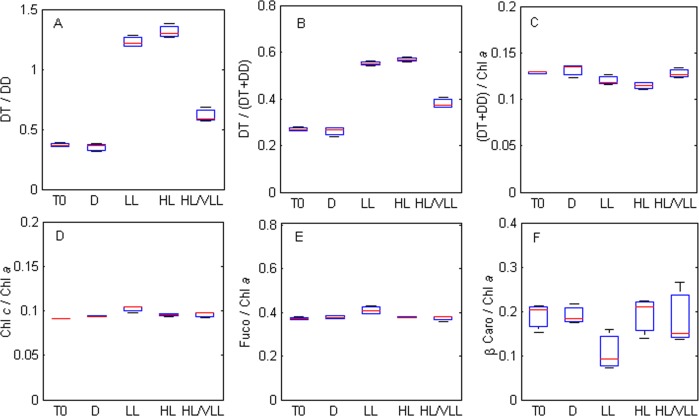
Comparison between pigment ratios (g g^-1^, experiment 1) measured before (T0) and after exposure to different light levels (D, LL, HL, HL/VLL). **A. Ratio diatoxanthin / diadinoxanthin. B. Ratio diatoxanthin / xanthophyll cycle pigments** (diadinoxanthin + diatoxanthin)**. C. Ratio xanthophyll cycle pigments / chlorophyll *a*. D. Ratio chlorophyll *c* / chlorophyll *a*. E. Ratio fucoxanthin / chlorophyll *a*. C. Ratio** β**-carotene / chlorophyll *a*.**

The source of *Haynesina germanica* pigments was clearly from ingested diatoms, with the typical diatom pigments Chl *a*, Fuco, Chl *c*, DD, DT and β-caro observed in freshly isolated specimens from the sediment (Fig 2). Initial pigment content was 6.08 ± 1.21 ng cell^-1^, with Chl *a* accounting for ~48%, Fuco for ~18%, β-caro for ~13%, the xanthophyll pigments (DD+DT) for ~6%, Chl *c* for ~4% and the sum of the degradation pigments, mainly fucoxanthin by-products (fuco-like pigment 1 and 2) for ~14%. Globally, *H*. *germanica* pigment profiles were similar throughout experiment 1; however, total pigment content was lower in condition HL/VLL compared to the other ones (Table 1). Only the photo-protective pigments, DD (P = 0.004) and DT showed significant differences between T0 and the light treatments (P = 0.0001, Fig 3 and Table 1). The ratio DT/DD was thus significantly different depending on the light treatments (F = 300, P < 0.001); at T0 at VLL the ratio DT/DD was equal to 0.37 ± 0.02 g g^-1^, after 30 minutes in the dark this ratio did not show any significant difference to T0 (0.35 ± 0.04 g g^-1^); whereas, it increased to 1.23 ± 0.05 and 1.35 ± 0.06 g g^-1^after 30 minutes of LL and HL exposition, respectively. It was also noteworthy that DT/DD decreased to 0.61 ± 0.06 g g^-1^ after the treatment HL:VLL (Fig 4A). A similar pattern was also found using the DT/ (DD+DT) ratio (Fig 4B, F = 273, P < 0.001). Significant differences were also observed between (DD+DT) /Chl *a* ratios (Fig 4C, F = 5.42, P = 0.01) with a significant decrease of the (DD+DT) /Chl *a* ratio observed under the two light treatments (LL and HL, Fig 4C) compared to the other conditions. The two light harvesting pigments Chl *c* and Fuco were also quantified but no significant differences were found under the different light treatment (Table 1); however, significant differences were found using the ratio Chl *c* / Chl *a* (Fig 4D, F = 8.82, P = 0.03); the ratio Chl *c* / Chl *a* increased under LL treatments; whereas, it was similar in the other conditions (Fig 4D). The ratio Fuco / Chl *a* was similar between all experimental treatments (Fig 4E, F = 1, P = 0.42). Finally, the ratio β-caro / Chl *a* was not different between conditions (Fig 4F, F = 2, P = 0.19).

**Table 1:** Chlorophyll, carotenoid and degraded pigments in *Haynesina germanica* (3 replicates of 100 specimens in each treatments) as a function of light treatments (T0, initial composition; D, Dark; LL, low light (25 μmol photons m^-2^ s^-1^); HL, high light (300 μmol photons m^-2^ s^-1^); HL/VLL, high light: very low light cycle (300:5 μmol photons m^-2^ s^-1^). Results from the ANOVA are presented, values are significantly different when p < 0.05 and then values with the same letter (a or b) are similar (Fisher, p > 0.05). (n = 3, Df = degree of freedom, DP = Degraded pigments which are mainly Fuco-likes pigments).

ng cell^-1^	Chl c	But-fuco	Fuco	Fuco-likes	DD	DT	Chl a	β-caro	DP	Total
T0	0.27 ± 0.03	0.02 ± 0.01	1.09 ± 0.12	0.84 ± 0.18	0.28 ± 0.03 b	0.10 ± 0.01 a	2.92 ± 0.32	0.56 ± 0.08	0.85 ± 0.20	6.08 ± 1.21
D	0.28 ± 0.01	0.04 ± 0.01	1.14 ± 0.01	0.91 ± 0.11	0.29 ± 0.01 b	0.10 ± 0.01 a	3.03 ± 0.06	0.58 ± 0.05	0.92 ± 0.10	6.40 ± 0.12
LL	0.35 ± 0.03	0.04 ± 0.01	1.38 ± 0.1	0.98 ± 0.07	0.18 ± 0.01 a	0.22 ± 0.01 b	3.37 ± 0.23	0.35 ± 0.07	1.02 ± 0.11	6.92 ± 0.65
HL	0.34 ± 0.01	0.04 ± 0.01	1.34 ± 0.03	1.04 ± 0.07	0.18 ± 0.06 a	0.23 ± 0.01 b	3.55 ± 0.07	0.68 ± 0.08	1.07 ± 0.07	7.42 ± 0.2
HL/VLL	0.25 ± 0.05	0.02 ± 0.01	0.95 ± 0.15	0.76 ± 0.21	0.20 ± 0.03 a	0.12 ± 0.02 a	2.54 ± 0.45	0.50 ± 0.19	0.78 ± 0.23	5.36 ± 1.76
ANOVA										
F (Df = 4)	2.52	1.4	3.38	1.96	7.83	22.04	2.17	1.24	1.85	1.87
p (α = 0.05)	0.1074	0.3013	0.0536	0.1770	0.004	0.0001	0.1457	0.3536	0.196	0.1928

### Experiment 2

*Haynesina germanica* had a mean maximal elongation of 315 ± 60 μm (SD, n = 60) and respective cytoplasmic biovolumes equal to 6.13 × 10^6^ ± 4.27 × 10^4^ μm^3^ (SD).

Initial pigment content was 27.35 ± 1.30 ng cell^-1^ with Chl *a* accounting for ~53%, Fuco for ~22%, the XCP for ~6%, Chl *c* for ~6% and the sum of the degradation pigments for ~12%. The β-carotene was below detection limit.

Between experiment 1 and experiment 2 a clear difference (P < 0.01) was found between total *H*. *germanica* pigment contents (Tables 1 and 2) with higher values in winter, i.e. 27.35 ± 1.30 ng cell^-1^ and lower in July, i.e. 6.08 ± 1.21 ng cell^-1^; however, pigment ratios remained stable with the exception of Fuco by-products (Fuco-like 1 and 2) of and of β-caro.

**Table 2:** Chlorophyll, carotenoid and degraded pigments in *Haynesina germanica* (3 replicates of 100 specimens in each treatments) as a function of time (days: 0, 5, 10, 15) and light treatments (L, light (50 μmol photons m^-2^ s^-1^); D, Dark (0 μmol photons m^-2^ s^-1^)). Results from the Multifactor ANOVA are presented, values from day 5 to 15 are significantly different when p < 0.05. (n = 3, Df = degree of freedom, DP = Degraded pigments which are mainly Fuco-likes pigments).

		ng cell^-1^	Chl *c*	But-fuco	Fuco	Fuco-likes	DD	DT	Chl *a*	DP	Total
T0		-	1.60 ± 0.06	0.06 ± 0.01	6.16 ± 0.21	1.85 ± 0.41	1.03 ± 0.05	0.55 ± 0.01	14.56 ± 0.76	3.40 ± 0.62	27.35 ± 1.30
5 days		Light	0.30 ± 0.03	0.03 ± 0.01	1.17 ± 0.14	1.09 ± 0.05	0.29 ± 0.01	0.24 ± 0.05	2.76 ± 0.43	1.69 ± 0.16	6.47 ± 1.04
5 days		Dark	0.44 ± 0.06	0.10 ± 0.02	1.75 ± 0.25	3.00 ± 0.89	0.19 ± 0.02	0.43 ± 0.06	3.72 ± 0.39	4.05 ± 1.20	10.64 ± 2.60
10 days		Light	0.23 ± 0.02	0.03 ± 0.01	0.91 ± 0.08	1.29 ± 0.48	0.17 ± 0.02	0.33 ± 0.04	2.25 ± 0.24	1.52 ± 0.51	5.44 ± 1.21
10 days		Dark	0.41 ± 0.12	0.11 ± 0.03	1.60 ± 0.47	4.28 ± 0.85	0.18 ± 0.05	0.46 ± 0.08	3.76 ± 1.05	4.47 ± 0.97	10.98 ± 4.05
15 days		Light	0.12 ± 0.01	0.02 ± 0.01	0.45 ± 0.01	0.90 ± 0.07	0.10 ± 0.01	0.21 ± 0.01	1.11 ± 0.11	1.03 ± 0.07	3.04 ± 0.26
15 days		Dark	0.30 ± 0.01	0.10 ± 0.01	1.18 ± 0.04	3.92 ± 0.71	0.13 ± 0.01	0.42 ± 0.03	2.81 ± 0.24	4.08 ± 0.72	9.01 ± 0.43
Multifactor ANOVA											
Time		F (df = 2)	5.5	0.1	5.1	2.35	8.8	1.4	4.2	0.8	0.70
(5 to 15 days)		p (α = 0.05)	0.012	0.9263	0.021	0.132	0.003	0.281	0.04	0.468	0.511
Light treatment		F (df = 1)	17	37.2	16.1	89.71	0.3	21.3	12.8	91.04	7.02
		p (α = 0.05)	0.001	<0.001	0.001	<0.001	0.582	<0.001	0.003	<0.001	0.019

**Fig 5:**
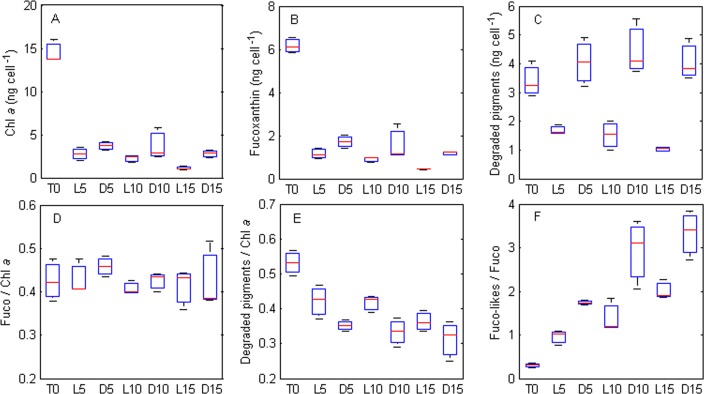
Comparison between pigments (ng cell^-1^) and pigment ratios (g g^-1^, experiment 2) measured before (T0) and after exposure to different light levels (D, L) and days (5, 10, 15). A. Chlorophyll *a*. B. Fucoxanthin. C. The sum of degraded pigments. D. Ratio fucoxanthin / chlorophyll *a*. E. Ratio sum of degraded pigments / chlorophyll *a*. F. Ratio sum of fucoxanthin by-products / fucoxanthin.

Total pigment content decreased drastically from 27.35 ± 1.30 ng cell^-1^ to 6.47 ± 1.04 and 10.64 ± 2.60 ng cell^-1^ after 5 days of exposure to light and dark treatments, respectively. The decrease between day 0 and day 5 was observed for most pigments, with the exception of 19'Butanoyloxyfucoxanthin and Fuco-likes (1 and 2) pigments. After the first 5 days of exposure to either light treatment, i.e. between days 5 to 15, total pigment content did not significantly changed (p > 0.05, Table 2). No differences as a function of time were also observed for: DT, the sum of degraded pigments and the fucoxanthin derivatives (Fuco-likes; Table 2 and Fig 5C). However, Chl *a* and *c* (Fig 5A and Table 2), Fuco (Fig 5B and Table 2) and DD slowly decreased over time (p > 0.05, Table 2) resulting in the stabilisation of Fuco/Chl *a* ratios (Fig 5D) and in the decrease of degraded pigments/Chl *a* ratios (Fig 5E). A decrease between dark and light treatment was also observed for most pigments with the exception of DD (Table 2). Additionally, the pigment ratio Fuco-likes/Fuco ratio (Fig 5F) increased over time (p < 0.05) and as a function of light treatment (p < 0.001), with higher values in the dark than under light (Fig 5D); whereas, the sum of degraded pigments/Chl *a* was only significantly affected by time (p < 0.05, Fig 5E).

## Discussion

Little is known about benthic foraminifera kleptoplast pigment content over time and seasons and pigment changes as a function of light. Furthermore, as *H*. *germanica* behaviour does not seem to be light driven [39] their photo-regulation capacity is likely to depend mainly on their kleptoplast physiological capabilities as for other kleptoplastic organisms [23, 41, 46]. We addressed this gap in current knowledge by investigating how *H*. *germanica* pigment content varied over time and light history, to determine if sequestered plastids might be capable of physiological photo-regulation via a functional xanthophyll cycle.

### Pigment composition of freshly sampled specimens

All pigments detected and quantified in *H*. *germanica* specimens were from diatom origin, i.e. six lipophilic pigments were detected, two chlorophylls (Chl *a*, Chl *c*) and four carotenoids (fucoxanthin and by-products, diadinoxanthin, diatoxanthin and β-carotene). These pigments profiles were close to those usually found in diatoms isolated from Bourgneuf bay mudflats [47–50] and confirmed previous *H*. *germanica* pigment composition results and kleptoplast origins [11, 18, 21]. Furthermore, as noticed by Knight and Mantoura [21] there was a clear variation in the cytoplasm colour between specimens, going from pale green to dark green, going from pale green to dark green. These individual differences added to size and bio-volume variations explained why we pooled 100 specimens for each HPLC sample and explained why total pigment varied between conditions (e.g. HL/VLL, Table 1). Average pigment content per cell also showed significant differences between specimens isolated in winter or summer. Only half (53%) of this difference can be attributed to differences in bio-volume (experiment 1, total pigment = 1.86 ± 0.37 fg μm^-3^ and experiment 2, total pigment = 4.46 ± 0.21 fg μm^-3^, P < 0.01), we thus suggest that the observed difference was mainly driven by different light availability at the two seasons, with higher pigment production and/or higher plastid assimilation linked to lower light intensity in the winter. Furthermore, pigment qualitative composition reflected *H*. *germanica* plastid light acclimation to low or high light availability according to the season; i.e. Fuco/Chl *a* ratio, which is a microphytobenthos photo-adaptation biomarker [51], varied between the two seasons at T0, being lower in the summer (July 0.37 ± 0.01 g g^-1^ (exp 1) and higher in December 0.43 ± 0.05 g g^-1^ (exp 2)). Also, the photoprotective pigment β-carotene levels were below the detection limit in the winter experiment, thus supporting the hypothesis that winter specimens were acclimated to lower light levels, similarly to the diatoms on which they fed. However, other physico-chemical parameters might affect their kleptoplast photoprotective mechanisms or pigment content such as temperature [52] or nitrogen requirements [49], requiring further experiments on benthic foraminifera physiology.

### Short term photo-acclimation (experiment 1)

*Haynesina germanica* lives mainly in the first millimeters of intertidal mudflat sediments [34–36, 53] and may thus be exposed to strong light variations during tidal periods ranging from zero in the sediment to high values (e.g > 2000 μmol photon m^-2^ s^-1^ [54, 55]) at the sediment surface. Microphytobenthos often use vertical migration into the sediment as a behavioral photo-regulation mechanism (e.g.[33]) whereby benthic diatoms escape saturating light intensities by moving a few microns deeper in the sediment matrix. Thus, if *H*. *germanica* is not sensitive to light [39] then we can hypothesise that the xanthophyll cycle might be an important short-term photoprotective mechanism for the diatom kleptoplasts incorporated in the foraminifera cytosol. This was supported by our experiments, i.e. there was an increase in DT/DD ratios when foraminifera were exposed to light that was reversed when the same foraminifera were exposed to VLL (Fig 4), showing that the XC was functional at least in short-term time scales (i.e. 30 min). To our knowledge, this is the first evidence of a functional xanthophyll cycle in kleptoplastic foraminifera and it is indicative of the presence of a short-term photo-protective mechanism protecting *H*. *germanica* kleptoplasts from photo-oxidative damages caused by excessive light.

The activation of the xanthophyll cycle under light exposure is therefore still functional after plastid assimilation in *H*. *germanica* cytoplasm. However, DT/ (DD+DT) ratio of *H*. *germanica* exposed to the HL:VLL cycle did not completely return to its initial state. It suggests that lumen acidification and subsequent diadinoxanthin de-epoxidase activation was not easily reversed in the kleptoplasts during the very low light cycle, thus still promoting a higher DT/ (DD+DT) ratio than in total darkness. Alternatively, no statistical differences between DT/ (DD+DT) ratio were observed between specimens exposed to LL (25 μmol photon m^-2^ s^-1^) and HL (300 μmol photon m^-2^ s^-1^), suggesting that at 25 μmol photon m^-2^ s^-1^ most of the DD was already converted in DT and thus that the light levels necessary to activate the XC were very low, i.e. < 25 μmol photon m^-2^ s^-1^ (LL) and maybe even as low as 5 μmol photon m^-2^ s^-1^ (VLL). This early de-epoxidation reaction activated at low light showed that the kleptoplasts react similarly to low-light adapted photosynthetic organisms, as they quickly increase their de-epoxidation state [56–58]. Furthermore, the small difference between the DT/(DD+DT) ratio at LL and HL light showed that at 25 μmol photon m^-2^ s^-1^ the kleptoplasts were already close to their maximal de-epoxidation sate. Interestingly, the maximal de-epoxidation state reached by foraminiferal kleptoplasts was high (0.55–0.6) for a short-term exposition compared to the diatoms on which they fed (0.3–0.5) [37, 50, 55]. This difference might be due to the kinetic of pigment degradation (see below), as the DT/(DD+DT) ratio was already high at T0 or under dark condition (0.25–0.3) compare to microphytobenthic species or other diatom species (<0.1) [50, 55]. It could also be that the pH was different (lower) in the foraminifera cytosol thus activating the XC enzymes quicker. In benthic foraminifera pH is known to vary between 5 to 9 [59], with high values (> 7–7.5) only found in the ultimate chamber during calcification process [59, 60]. A chamber that do not contain kleptoplasts. Thus, the difference between DT/(DD+DT) in the dark and under high light was in the same range of order than what was observed in benthic diatoms [37, 50, 55], highlighting the fact that the kleptoplasts keep some of the photophysiological traits of the species on which they feed.

### Pigment changes over time (experiment 2)

To follow how kleptoplast pigment composition changed over time, we surveyed the pigment content and composition at two light regimes (0 and 50 μmol photon m^-2^ s^-1^) of starved *H*. *germanica* specimens. The effect of food deprivation showed a biphasic decrease of kleptoplast total pigment content. A rapid decrease was observed during the first five starvation days, which was followed by a slow decrease over the remaining ten days. We suggest that this trend is the result of two different processes. The rapid initial decrease would correspond to chloroplasts that were ingested but not integrated as kleptoplasts and were thus in a process of cellular digestion and the latter slower phase would correspond to true kleptoplasts that were slowly being degraded with time. This would also suggest that only some diatom chloroplasts are capable kleptoplasts. Similar results were observed in other kleptoplastic organisms, e.g. in some sea slugs species a fast chloroplast degradation was observed after sampling followed by a second period were some chloroplasts remained up to 15 days [61]. However, using HPLC analysis it is not possible to identify chloroplasts from different diatom species thus this hypothesis will require further analysis.

Kleptoplast pigment composition did not change much throughout the 15 days experiment with an increase of some ratios such as Fuco-likes/Fuco suggesting that kleptoplasts were slowly degrading and thus producing fucoxanthin by-products (mainly Fuco-like 1 and 2). Interestingly, the observation that Fuco/Chl *a* ratios were stable and that Fuco-likes/Fuco and (DD+DT)/Chl *a* ratio increased over time suggests a slower degradation of carotenoids compared to chlorophylls. Similar observations were made with other kleptoplastic species such as sacoglossan sea slugs *Elysia veridis* and *Thuridilla hopei* [24, 62]. Furthermore, studies on sacoglossan speculated that this slower carotenoid degradation rate might be a selective process [24, 62] as carotenoids are known to be beneficial for animal health (e.g., photoprotection, camouflage, signalling and antioxidative activities [63]). In the present study (experiment 2) higher light levels also induced higher pigment decay, underlying the importance of taking into account the light history of the kleptoplastic organism in any study dealing with kleptoplasts. Furthermore, for DT and DD, under prolonged darkness more than threefold more DT than DD was observed at the end of the experiment. This might be the result of diatoms de-epoxidation in the dark driven by the chlororespiratory proton gradient [45]. However, it is possible also that this is a side effect of lower DT degradation in the dark, although the DT/Chl *a* ratios seem very similar between treatments. A similar increase of Zeaxanthin in the dark was also observed in the Jesus et al. [23] study with *Elysia timida*, thus it is also possible that heterotrophic cells in darkness exhibit lower pH inducing an activation of the XC in the dark different from algal cells.

Kleptoplast functionality and pigment content are necessarily linked. In a previous experiment we assessed kleptoplast functionality as function of light and time (6 days) using starved *H*. *germanica* specimens [18]. In darkness, *H*. *germanica* photosystem II maximum quantum efficiency (*Fv/Fm*) slowly decreased from 0.65 to 0.55 within a week; whereas, a quick decrease to 0.2 was found under light exposure (70 μmol photon m^-2^ s^-1^). In the present study the difference between pigment content was significant but not so large between specimens kept in the darkness or under light; however, this difference can be explained as *Fv/Fm* measured using pulse amplitude fluorometry can stay high even if only a reduced number of functional kleptoplasts remain active. Interestingly, similar observations were previously made with sacoglossans, suggesting similarities between kleptoplast functionality in different organisms [23, 62, 64].

## Conclusion

This study measured *H*. *germanica* kleptoplasts photo-regulation capacities and monitored pigment composition, over time and with different light histories. The main diatom lipophilic pigments were detected: chlorophylls (Chl *a* and *c*) and different carotenoids (fucoxanthin and by-products, diadinoxanthin, diatoxanthin and β-carotene), similarly to diatom pigment profiles isolated at the same sampling place. Furthermore, the current study showed that there were seasonal differences between pigments from specimens isolated in winter or summer, with higher pigment contents in winter specimens, indicating that *H*. *germanica* acclimatize to ambient light levels or keep the diatom plastids light acclimation state. *Haynesina germanica* xanthophyll cycle was found to be functional with DT/(DT+DD) ratios increasing under light exposure and reversing back in darkness or at very low light. This suggests that *H*. *germanica* kleptoplasts retain some of their physiological photo-regulation mechanisms active after being incorporated in foraminifera cytosol. To our knowledge, this is the first evidence of a functional xanthophyll cycle in kleptoplastic foraminifera and it is indicative of the presence of a short-term photo-protective mechanism protecting *H*. *germanica* kleptoplasts from photo-oxidative damages caused by excessive light. Finally, the pigment survey suggests that *H*. *germanica* preserved some chloroplasts over a longer time than others and that pigment content is influenced by light history.
